# Supplementary material for: Deficiency of exopolysaccharides and O-antigen makes Halomonas bluephagenesis self-flocculating and amenable to electrotransformation
Source: Commun Biol. 2022 Jun 24;5:623. doi: 10.1038/s42003-022-03570-y (PMC9232590; doi:10.1038/s42003-022-03570-y)
Supplement: Supplementary file 2 — Supplementary Information [file 42003_2022_3570_MOESM2_ESM.pdf]

Supplementary materials

Deficiency of exopolysaccharides and O-antigen makes *Halomonas bluephagenesis* self-flocculating and amenable to electrotransformation

Authors:

Tong Xu[a], Junyu Chen[a], Ruchira Mitra[ab], Lin Lin[ac], Zhengwei Xie[d], Guo-Qiang Chen[*e], Hua Xiang[*ab], Jing Han[*ab]

Affiliations:

[a]State Key Laboratory of Microbial Resources, Institute of Microbiology, Chinese Academy of Sciences, Beijing 100101, People's Republic of China

[b]International College, University of Chinese Academy of Sciences, 100049, Beijing, People's Republic of China

[c]College of Life Science, University of Chinese Academy of Sciences, 100049, Beijing, People's Republic of China

[d]Peking University International Cancer Institute, Health Science Center, Peking University, Beijing, People's Republic of China

[e]Center for Synthetic and Systems Biology, School of Life Sciences, Tsinghua University, Beijing, People's Republic of China

[*]Corresponding authors: Guo-Qiang Chen, Hua Xiang and Jing Han

E-mail: chengq@mail.tsinghua.edu.cn (G.-Q. Chen), xiangh@im.ac.cn (H. Xiang) or hanjing@im.ac.cn (J. Han)

Membrane permeability assay

NPN is a hydrophobic molecule fluorescing strongly in phospholipid but weakly in aqueous solutions. Intact outer membrane has the ability to exclude external hydrophobic NPN. But the disturbed integrity of outer membrane enables NPN entry into phospholipid of cell membrane and causes fluorescence change. Thus, the 1-N-phenylnaphthylamine (NPN) method has been used to assess outer membrane permeability[1]. *H. bluephagenesis* cells cultured overnight in LB60 medium were washed and resuspended 1 M NaCl solution. The value of OD_{600} was adjusted to 0.7 with the same buffer. Then, NPN uptake assays were performed as described previously[1].

Transmission electron microscopy

For transmission electron microscopy (TEM), cells cultured in 60MMG medium for 36 h were harvested by centrifugation at 5000 g for 3 min, and then washed with PBS buffer (pH 7.2). Subsequently, the cells were fixed with 1 ml of 2% glutaraldehyde dissolved in PBS buffer (pH 7.2) and kept at room temperature for 30 min and then stayed at 4°C overnight. After centrifugation at 5000 g for 3 min, the cell pellets were fixed with 1 ml of 1.0% osmium tetroxide dissolved in PBS buffer and kept for 2 h and subsequently washed three times with PBS buffer. The sample preparation was performed according to the method described by Tian et al.[2]. Microscopy imaging were carried out using a Philips JEOL-1400 electron microscope.

Supplementary Table 1. List of gene clusters deleted in *H. bluephagenesis* TD1.0 and the essential genes located in the regions between these clusters

Cluster	Length	Accession number	Protein name
PS1	16861 bp	WP_039868450.1	**Capsular polysaccharide export system protein KpsC**
		WP_009721844.1	**Capsular polysaccharide export system protein KpsS**
		WP_009721843.1	**Short-chain dehydrogenase**
		WP_039868553.1	**Capsular polysaccharide biosynthesis protein WcbQ**
		WP_009721841.1	**Capsular polysaccharide biosynthesis fatty acid synthase WcbR**
		WP_009721840.1	7-keto-8-aminopelargonate synthetase
		WP_009721839.1	Transcription elongation factor/antiterminator RfaH
		WP_009721838.1	Nucleoside diphosphate kinase regulator
		WP_009721837.1	NADP-specific glutamate dehydrogenase GdhA
Essential genes between PS1 and PS2		WP_009721834.1	UTP-glucose-1-phosphate uridylyltransferase
		WP_009721827.1	glutamine-fructose-6-phosphate transaminase
		WP_009721826.1	3'(2'),5'-bisphosphate nucleotidase CysQ
PS2	22861 bp	WP_009721819.1	**Glycosyl transferase group 1**
		WP_009721818.1	**Glycosyl transferase group 1**
		WP_009721817.1	**UDP-N-acetyl-D-mannosamine dehydrogenase**
		WP_009721816.1	**UDP-N-acetylglucosamine 2-epimerase**
		WP_009721815.1	Hypothetical protein
		WP_009721814.1	Hypothetical protein
		WP_009721813.1	Sulfotransferase family protein
		WP_009721812.1	Virginiamycin a acetyltransferase
		WP_009721811.1	**UDP-N-acetylglucosamine C-6 dehydrogenase**
		WP_009721810.1	Hypothetical protein
		WP_009721809.1	Chromosome segregation protein smc
		WP_009721808.1	**Glycosyl transferase group 1**
		WP_009721807.1	**dTDP-4-dehydrorhamnose 3,5-epimerase**
Essential genes		WP_009721806.1	Glucose-1-phosphate thymidylyltransferase RfbA

between PS2 and PS4		WP_009721804.1	dTDP-glucose 4,6-dehydratase
PS4	14851 bp	WP_009721803.1	**Capsular polysaccharide ABC transporter**
		WP_009721802.1	RNA procession exonuclease
		WP_009721798.1	**Capsular polysaccharide export system inner membrane protein KpsE**
		WP_009721797.1	**Capsular polysaccharide ABC transporter, permease protein KpsM**
		WP_009721796.1	**Capsular polysaccharide export system periplasmic protein KpsD**
		WP_009721795.1	Hypothetical protein
		WP_009721794.1	**O-antigen ligase**
		WP_009721793.1	**UDP-phosphate galactose phosphotransferase**
		WP_009721792.1	**Glycosyl transferase group 1**
		WP_009721791.1	**Glycosyl transferase family 28**
		WP_009721790.1	**Polysaccharide biosynthesis protein**
		WP_009721789.1	Hypothetical protein
		WP_143759699.1	**Lipopolysaccharide biosynthesis protein**
PS3	16379 bp	WP_009722803.1	**dTDP-4-dehydrorhamnose 3,5-epimerase**
		WP_009722804.1	**CDP-glucose 4,6-dehydratase RfbG**
		WP_009722805.1	**Glucose-1-phosphate cytidylyltransferase RfbF**
		WP_009722806.1	**Chondroitin polymerase**
		WP_009722807.1	Flagellar biosynthesis protein FliC
		WP_009722808.1	Flagellar biosynthesis protein FliC
		WP_009722809.1	Flagellar hook-associated protein FliD
		WP_009722811.1	Flagellar hook-associated protein FliD
		WP_009722812.1	Hypothetical protein
		WP_009722813.1	Serine hydrolase
		WP_083817051.1	YqhA family protein
		WP_009722816.1	**Exopolysaccharide biosynthesis protein**

Note: The genes involved in the synthesis of EPSs and precursor sugars are in bold.

Supplementary Table 2. Differential gene expression analysis of ΔPS124 *vs.* TD1.0.

Gene_ID	log_2(Fold Change)	Gene Name	Protein Name
Up-regulated			
GME_09284	2.799568	*hppD*	4-hydroxyphenylpyruvate dioxygenase; Hemolysin VllY
GME_00855	1.28826	*hyp*	hypothetical protein
GME_00470	1.214056	*gabD*	succinate-semialdehyde dehydrogenase [NADP+]
GME_19262	1.507446	*fhaB*	putative large exoprotein involved in heme utilization or adhesion of ShlA/HecA/FhaA family; filamentous hemagglutinin
GME_00475	1.037963	*doeD*	gamma-aminobutyrate: alpha-ketoglutarate aminotransferase; L-2,4-diaminobutyrate transaminase; uncharacterized aminotransferase y4uB
Down-regulated			
GME_05380	-3.36565	*nirB2*	nitrite reductase [NAD(P)H] large subunit;nitrite reductase (NADH) large subunit
GME_14108	-2.93316	td01_2366	rhodanese-related sulfurtransferase; sulfurtransferase
GME_05375	-2.89649	*nirB1*	nitrite reductase [NAD(P)H] large subunit; nitrite reductase (NADH) large subunit
GME_14113	-2.7546	td01_2367	putative peroxiredoxins
GME_16605	-2.59799	td01_7	hypothetical protein
GME_05385	-2.33686	*nasA*	assimilatory nitrate reductase large subunit; assimilatory nitrate reductase catalytic subunit; nitrate reductase
GME_16620	-2.31436	td01_10	hypothetical protein
GME_16615	-2.25947	td01_9	hypothetical protein
GME_15800	-2.25313	cydA2	cytochrome d ubiquinol oxidase subunit I
GME_16952	-1.95366	td01_197	uncharacterized ABC1 family protein; ubiquinone biosynthesis protein ubib

GME_05455	-1.89935	*narK4*	nitrate/nitrite transporter; MFS transporter, NNP family, nitrate/nitrite transporter; High affinity nitrate transporter 2.5
GME_02355	-1.87401	*gloB*	Zn-dependent hydrolases, including glyoxylases; Glyoxylase B2
GME_15795	-1.80722	*cydB*	cytochrome d ubiquinol oxidase subunit II
GME_05370	-1.80248	*nirD*	nitrite reductase [NAD(P)H] small subunit; nitrite reductase (NADH) small subunit
GME_05450	-1.78117	td01_2858	response regulator NasT; response regulator receiver protein
GME_05275	-1.76916	td01_2823	hypothetical protein
GME_02275	-1.6769	td01_660	T1SS secreted agglutinin RTX;hemolysin, plasmid
GME_02149	-1.63517	td01_693	cryptochrome; deoxyribodipyrimidine photolyase
GME_16585	-1.6329	td01_3	phage repressor protein
GME_13510	-1.6201	td01_2085	putative transcriptional regulator
GME_16575	-1.61419	td01_3988	hypothetical protein
GME_02360	-1.61145	td01_644	permeases of the major facilitator superfamily
GME_10736	-1.54561	*cydA1*	cytochrome d ubiquinol oxidase subunit I
GME_14995	-1.52868	td01_2549	FAD-dependent pyridine nucleotide-disulphide oxidoreductase
GME_05390	-1.52593	*cysG1*	uroporphyrinogen-III methyltransferase; uroporphyrin-III C-methyltransferase; precorrin-2 dehydrogenase; sirohydrochlorin ferrochelatase; cysG
GME_02370	-1.48923	td01_642	gene II and X proteins
GME_02365	-1.48174	td01_643	probable transmembrane protein; UPF0394 membrane protein PD_1892

GME_08274	-1.46206	*mmsA3*	methylmalonate-semialdehyde dehydrogenase; malonate-semialdehyde dehydrogenase (acetylating)
GME_04417	-1.45934	*vanR*	transcriptional regulator, GntR family; GntR family transcriptional regulator, vanillate catabolism transcriptional regulator
GME_13175	-1.44956	td01_2154	hypothetical protein
GME_02695	-1.44909	td01_572	glucose-1-phosphate thymidylyltransferase
GME_13515	-1.44537	td01_2084	oxidoreductase, short-chain dehydrogenase/reductase family
GME_05855	-1.43708	td01_2944	threonine dehydrogenase and related Zn-dependent dehydrogenases
GME_10871	-1.43247	td01_3679	SOS-response repressor and protease LexA; LexA family transcriptional regulator
GME_08504	-1.4303	td01_1502	hypothetical protein
GME_02500	-1.39125	*msrB1*	peptide methionine sulfoxide reductase MsrB
GME_15000	-1.38826	td01_2550	sulfate permease; sulfate permease, SulP family; sulfate: proton symporter
GME_08279	-1.3769	td01_1456	omega-amino acid--pyruvate aminotransferase; beta-alanine--pyruvate transaminase
GME_18605	-1.35932	*uspA*	universal stress protein family;universal stress protein A;universal stress protein uspA
GME_13520	-1.34178	td01_2083	amine oxidase, flavin-containing
GME_05850	-1.31441	td01_2943	CDP-alcohol phosphatidyltransferase
GME_02690	-1.3087	*rfbD1*	dTDP-4-dehydrorhamnose reductase
GME_02264	-1.30419	td01_670	T1SS secreted agglutinin RTX
GME_11602	-1.30106	td01_3530	flavin-containing monooxygenase; putative flavoprotein involved in K^+ transport

GME_11407	-1.28712	td01_3570	poly(3-hydroxyalkanoate) synthetase
GME_11557	-1.28396	ompW2	outer membrane protein W precursor
GME_15305	-1.27978	td01_3729	hypothetical protein
GME_04697	-1.26861	*lexA*	SOS-response repressor and protease LexA; repressor LexA
GME_16632	-1.26622	td01_12	recombinase
GME_17242	-1.2309	*frdA*	succinate dehydrogenase flavoprotein subunit; fumarate reductase flavoprotein subunit
GME_09841	-1.21899	td01_1781	hypothetical protein
GME_05860	-1.21841	td01_2945	PTPS-like type 4; 6-pyruvoyl tetrahydropterin synthase
GME_16002	-1.20762	td01_3871	putative lipoprotein precursor
GME_05560	-1.20309	td01_2879	inosine-5'-monophosphate dehydrogenase
GME_14710	-1.18385	td01_2489	membrane-bound metal-dependent hydrolase; inner membrane protein
GME_01699	-1.18239	td01_788	transcriptional regulator, lysr family
GME_06430	-1.17611	td01_3059	transglycosylase-associated protein
GME_13903	-1.16585	td01_2002	short chain dehydrogenase
GME_08169	-1.15686	td01_1433	possible pyrimidine permease in reductive pathway; nucleobase:cation symporter-1, NCS1 family; allantoin permease
GME_19284	-1.15328	td01_670	T1SS secreted agglutinin RTX
GME_18670	-1.14573	td01_3315	iron-sulfur cluster regulator IscR; rrf2 family transcriptional regulator
GME_02144	-1.12598	td01_694	hypothetical protein
GME_14715	-1.12156	td01_2490	CopG protein;metal-binding protein
GME_04647	-1.11843	*recA*	RecA protein; recombination protein RecA; recombinase RecA
GME_04852	-1.11391	td01_2738	hypothetical protein
GME_18665	-1.10814	td01_3315	iron-sulfur cluster regulator IscR; rrf2 family transcriptional regulator

GME_16277	-1.10166	*scoA*	succinyl-CoA:3-ketoacid-coenzyme A transferase subunit A
GME_03777	-1.09683	td01_347	hypothetical protein
GME_17467	-1.08513	td01_86	hypothetical protein
GME_07061	-1.07343	*elaB*	ElaB protein
GME_00615	-1.06908	*leuA*	2-isopropylmalate synthase; 2-isopropylmalate synthase 2
GME_13490	-1.0651	td01_2089	cell division inhibitor
GME_15785	-1.05195	*ybbA*	AttE component of AttEFGH ABC transport system; putative ABC transport system ATP-binding protein; Uncharacterized ABC transporter ATP-binding protein YbbA
GME_06839	-1.04954	*aroC*	chorismate synthase
GME_00885	-1.03846	*proV*	glycine betaine ABC transport system, ATP-binding protein OpuAA; glycine betaine/proline transport system ATP-binding protein
GME_13525	-1.0329	td01_2082	hypothetical protein
GME_17923	-1.02916	td01_1162	autotransporter adhesin; adhesin yada precursor
GME_16342	-1.02655	*pma*	Ca ion P-type ATPase; Ca2+-transporting ATPase; Cation-transporting ATPase pma1
GME_05865	-1.02607	td01_2946	glycosyl transferase, group 1
GME_05715	-1.00787	*yflS*	2-oxoglutarate/malate translocator; divalent anion: Na^+ symporter, DASS family; C4-dicarboxylate ABC transporter
GME_17682	-1.00049	*gyrA*	DNA gyrase subunit A

Supplementary Table 3. Strains and plasmids used in this study.

Name	Characteristics	Description
**Strains**		
E. coli S17-1	Conjunction donor, harbors the *tra* genes in the genome; *proA*, *thi-1*	[3]
E. coli DH5α	For plasmid construction, F^-, φ8 *lacZ*ΔM15, Δ(*lacZYA-argF*) U169, *deoR*, *endA1*, *recA1*, *hsdR17*(rk$^-$, mk$^-$), *supE44*, λ-, *thi-1*, *gyrA96*, *relA1*, *phoA*	TsingKe Biological Technology, China
H. bluephagenesis TD1.0	The RNAP module of MmP1 was integrated into the chromosome of *H. bluephagenesis* TD01	[4]
ΔPS1	TD1.0 ΔPS1 cluster	This study
ΔPS2	TD1.0 ΔPS2 cluster	This study
ΔPS3	TD1.0 ΔPS3 cluster	This study
ΔPS12	TD1.0 ΔPS1 cluster, ΔPS2 cluster	This study
ΔPS124	TD1.0 ΔPS1 cluster, ΔPS2 cluster, ΔPS4 cluster	This study
ΔPS1234	TD1.0 ΔPS1 cluster, ΔPS2 cluster, ΔPS4 cluster, ΔPS3 cluster	This study
ΔPS1234Δ50k	TD1.0 ΔPS1 cluster, ΔPS2 cluster, ΔPS4 cluster, ΔPS3 cluster, Δ50 kb of flagellar cluster	This study
ΔPS124Δ*fhaB*	ΔPS124, Δ*fhaB*	This study
ΔPS124Δ*hppD*	ΔPS124, Δ*hppD*	This study
ΔPS124Δ*hyp*	ΔPS124, Δ*hyp*	This study
ΔPS124Δ*hppD*Δ*hyp*Δ*fhaB*	ΔPS124, Δ*hppD*, Δ*hyp*, Δ*fhaB*	This study
TD1.0ΔO-antigen	TD1.0 ΔO-antigen cluster	This study
**Plasmids**		
pSEVA321	p321, RK2 replication origin, containing the *oriT* sequence for conjugate transformation, an expression vector in *H. bluephagenesis* TD01 strain, Cm^R. The lowest copy number plasmid used in this study	[5]
pSEVA241	pRO1600 / ColE1 replicon, containing the *oriT* sequence for conjugate transformation, an expression vector in *H. bluephagenesis* TD01 strain, Km^R and Sp^R. The highest copy number plasmid used in this study	[6]

pSEVA341	pRO1600/ColE1 replication origin, *oriT*, an expression vector in *Halomonas* TD strain, Cm^R	[5]
pQ08	pSEVA321 derivative, *Streptococcus pyogenes cas9*, Cm^R	[6]
p1g20k	pSEVA241 derivative, single sgRNA (20-kb flagella), H1 and H20 donor, $\underline{Km}^R$ and Sp^R	This study
p2g20k	pSEVA241 derivative, double sgRNAs (20-kb flagella), H1 and H20 donor, Km^R and Sp^R	This study
p2g30k	pSEVA241 derivative, double sgRNAs (30-kb flagella), H1 and H30 donor, Km^R and Sp^R	This study
p2g40k	pSEVA241 derivative, double sgRNAs (40-kb flagella), H1 and H40 donor, Km^R and Sp^R	This study
p2g50k	pSEVA241 derivative, double sgRNAs (50-kb flagella), H1 and H50 donor, Km^R and Sp^R	This study
p2gPS1	pSEVA241 derivative, double sgRNAs (PS1), PS1H1 and PS1H2 donor, Km^R and Sp^R	This study
p2gPS2	pSEVA241 derivative, double sgRNAs (PS2), PS2H1 and PS2H2 donor, Km^R and Sp^R	This study
p2gPS3	pSEVA241 derivative, double sgRNAs (PS3), PS3H1 and PS3H2 donor, Km^R and Sp^R	This study
p2gPS4	pSEVA241 derivative, double sgRNAs (PS4), PS4H1 and PS4H2 donor, Km^R and Sp^R	This study
p2gfhaB	pSEVA241 derivative, double sgRNAs (*fhaB*), fhaBH1 and fhaBH2 donor, Km^R and Sp^R	This study
p1ghppD	pSEVA241 derivative, single sgRNA (*hppD*), hppDH1 and hppDH2 donor, Km^R and Sp^R	This study
p1ghyp	pSEVA241 derivative, single sgRNA (*hyp*), hypH1 and hypH2 donor, Km^R and Sp^R	This study
p1gprofhaB	pSEVA241 derivative, single sgRNA (promoter region of *fhaB*), profhaBH1 and profhaBH2 donor, insert *lac*	This study

	promoter instead of promoter of *fhaB*, *Km*^{R} and *Sp*^{R}	
pSEVA321-Part1	pSEVA321 derivative, Part1 of PS4 cluster were subcloned into pSEVA321.	This study
pSEVA321-Part2	pSEVA321 derivative, Part2 of PS4 cluster were subcloned into pSEVA321.	This study
pSEVA321-Part3	pSEVA321 derivative, Part3 of PS4 cluster were subcloned into pSEVA321.	This study
p1gO-antigen	pSEVA241 derivative, single sgRNA (O-antigen), O-antigenH1 and O-antigenH2 donor, *Km*^{R} and *Sp*^{R}	This study

Supplementary Table 4. Primers used in this study.

Name	Sequence	Description
pSEVA241F	agccgtcgtgactgggaaaa	pSEVA241 linear plasmid for Gibson assembly
pSEVA241R	taccgagctcgaattcgcgc	
sgRNAF	gcgcgaattcgagctcggta	amplification of synthetic sgRNA
sgRNAR	gcattagctgcacatgcagt	
flagella H1F	actgcatgtgcagctaatgccacgttgtttgcctcatcgt	flagellar H1 homology arm
flagella H1R	aagattcgaattggccatgcgccaatcctcgttcgctgag	
flagella H20F	tggccaattcgaatcttaggtgcattacctgtgtcatcaa	flagellar H20 homology arm
flagella H20R	ttttcccagtcacgacggctcccaggaagcgttggtcgac	
flagella H30F	tggccaattcgaatcttaggaagataaaccattctcacgc	flagellar H30 homology arm
flagella H30R	ttttcccagtcacgacggctcggctagctggggagcataa	
flagella H40F	tggccaattcgaatcttaggcaccccattacgctataaat	flagellar H40 homology arm
flagella H40R	ttttcccagtcacgacggctatcggcattttgtttcaccg	
flagella H50F	tggccaattcgaatcttaggacagtgtttagccgtgaggc	flagellar H50 homology arm
flagella H50R	ttttcccagtcacgacggctatatcatcacgtacataggt	
flagella VF	ccttcacgaatagcttcgcg	verification of flagellar cluster deletion
flagella VWTR	ctcattgcggaactagcaat	
flagella V20kR	gggcgagattctcaacctga	
flagella V30kR	aagtcggcaagggcaatctt	
flagella V40kR	taccgaggcattgagtgcca	
flagella V50kR	cgtaggtagccatcgtacga	
PS1 H1F	actgcatgtgcagctaatgctaattagcgttctattcggg	upstream homology arm of PS1
PS1 H1R	cctaagattcgaattggccatgcgaaagagccgaatggtgtca	
PS1 H2F	gcatggccaattcgaatcttaggaaacaggcaccccttttatag	downstream homology arm of PS1
PS1 H2R	ttttcccagtcacgacggctatagtgtagccttgatatct	
PS2 H1F	actgcatgtgcagctaatgccaacgttatcagctaaatat	upstream homology arm of PS2
PS2 H1R	cctaagattcgaattggccatgctgtttttaaagttctagaat	
PS2 H2F	gcatggccaattcgaatcttaggatctaaaccgtaagaggtga	downstream homology arm of PS2
PS2 H2R	ttttcccagtcacgacggctaattattacagcgagttgag	
PS4 H1F	actgcatgtgcagctaatgctcggtgacgaacgtgatcag	upstream homology arm of PS4
PS4 H1R	cctaagattcgaattggccatgctgccagcgttgatgagatag	
PS4H2 F	gcatggccaattcgaatcttaggacactctctctcattcctca	downstream homology arm of PS4
PS4H2 R	ttttcccagtcacgacggcttgaagaaggcaacgtagttg	
PS3 H1F	actgcatgtgcagctaatgcaaatctggctttattccagc	

PS3 H1R	cctaagattcgaattggccatgcactgtcggcattgtgcaaca	upstream homology arm of PS3
PS3 H2F<br>PS3 H2R	gcatggccaattcgaatcttaggtggcgttttggcgctccatg<br>ttttcccagtcacgacggctaaaaaccgacactttggtcg	downstream homology arm of PS3
FhaB H1F<br>FhaB H1R	actgcatgtgcagctaatgccacaggaaaataccaagggg<br>cgcccaacgtgccgcttctttgttttctcgtatcaaaatt	upstream homology arm of *fhaB*
FhaB H2F<br>FhaB H2R	aattttgatacgagaaaacaaagaagcggcacgttgggcg<br>ttttcccagtcacgacggctattgcgtatctttcgaaccc	downstream homology arm of *fhaB*
HppD H1F<br>HppD H1R	actgcatgtgcagctaatgcccgaccctgcatatctatcg<br>tcttgttattcagcgttttacgctatctctcctattattg	upstream homology arm of *hppD*
HppD H2F<br>HppD H2R	caataataggagagatagcgtaaaacgctgaataacaaga<br>ttttcccagtcacgacggctgggcggtctacttgagctac	downstream homology arm of *hppD*
Hyp H1F<br>Hyp H1R	actgcatgtgcagctaatgccagcatcgtcagcatggctt<br>catcaatgaggtttctttctctctgtttatgacaaccctg	upstream homology arm of *hyp*
Hyp H2F<br>Hyp H2R	cagggttgtcataaacagagagaaagaaacctcattgatg<br>ttttcccagtcacgacggctcagtggaagacgctcgtcga	downstream homology arm of *hyp*
proH1F<br>proH1R	actgcatgtgcagctaatgctaactacaacagcgaaaaag<br>ttatccgctcacaattccacacaacatacgagccggaagcataaagtgtaaagtcacaatacatatatataa	upstream homology arm of promoter region of *fhaB*
proH2F<br>proH2R	cgtatgttgtgtggaattgtgagcggataacaatttcacacaggaaacagcttttcagtatctggaaaagga<br>ttttcccagtcacgacggctatgacgctcactccctgagc	downstream homology arm of promoter region of *fhaB*
PS4F1<br>PS4R1	gcgcgaattcgagctcggtaactatctcatcaacgctggc<br>aggtcgactctagaggatccggcttggccacttttgaaaa	Part1 of PS4 cluster
PS4F2<br>PS4R2	gcgcgaattcgagctcggtataggcacggcgttagatttc<br>aggtcgactctagaggatcccctgctatgagtcaaagttg	Part2 of PS4 cluster
PS4F3<br>PS4R3	gcgcgaattcgagctcggtactaacaaagcgatgccattg<br>aggtcgactctagaggatccgaagaaagtcggggtaaacc	Part3 of PS4 cluster
O-antiH1F<br>O-antiH1R	agccgtcgtgactgggaaaactttacttggtagctagcgt<br>tgaggaatgagagagagtgtgatcccgtgatgaaaaaagc	upstream homology arm of O-antigen
O-antiH2F	gcttttttcatcacgggatcacactctctctcattcctca	

O-antiH2R	tccaagactagtcgccagggtgaagaaggcaacgtagttg	downstream homology arm of O-antigen

Supplementary Table 5. Sequences of synthetic DNA used in this study.

**Name**	**Sequence**	**Description**
20k1sgRNA	gcgcgaattcgagctcggtattgacagctagctcagtcctaggtataatactagtgccgttttaagtcagtcactgttttagagctagaaatagcaagttaaaataaggctagtccgttatcaacttgaaaaagtggcaccgagtcggtgctttttttgaacccgggatgactgcatgtgcagctaatgc	single sgRNA for 20 kb of flagellar cluster
20k2sgRNA	gcgcgaattcgagctcggtattgacagctagctcagtcctaggtataatactagtccaagcgcgtcacgacgtgtgttttagagctagaaatagcaagttaaaataaggctagtccgttatcaacttgaaaaagtggcaccgagtcggtgctttttttgaacccgggatgttgacagctagctcagtcctaggtataatactagtgactaacgcgctgcgttgttgttttagagctagaaatagcaagttaaaataaggctagtccgttatcaacttgaaaaagtggcaccgagtcggtgctttttttgaacccgggatgactgcatgtgcagctaatgc	double sgRNA for 20 kb of flagellar cluster
30k2sgRNA	gcgcgaattcgagctcggtattgacagctagctcagtcctaggtataatactagtccaagcgcgtcacgacgtgtgttttagagctagaaatagcaagttaaaataaggctagtccgttatcaacttgaaaaagtggcaccgagtcggtgctttttttgaacccgggatgttgacagctagctcagtcctaggtataatactagtccagcccatgattatgtttggttttagagctagaaatagcaagttaaaataaggctagtccgttatcaacttgaaaaagtggcaccgagtcggtgctttttttgaacccgggatgactgcatgtgcagctaatgc	double sgRNA for 30 kb of flagellar cluster
40k2sgRNA	gcgcgaattcgagctcggtattgacagctagctcagtcctaggtataatactagtccaagcgcgtcacgacgtgtgttttagagctagaaatagcaagttaaaataaggctagtccgttatcaacttgaaaaagtggcaccgagtcggtgctttttttgaacccgggatgttgacagctagctcagtcctaggtataatactagtgtgtggtgttcggtatgccagttttagagctagaaatagcaagttaaaataaggctagtccgttatcaacttgaaaaagtggcaccgagtcggtgctttttttgaacccgggatgactgcatgtgcagctaatgc	double sgRNA for 40 kb of flagellar cluster
50k2sgRNA	gcgcgaattcgagctcggtattgacagctagctcagtcctaggtataatactagtccaagcgcgtcacgacgtgtgttttagagctagaaatagcaagttaaaataaggctagtccgttatcaacttgaaaaagtggcaccgagtcggtgctttttttgaacccgggatgttgacagctagctcagtcctaggtataatactagtttagccggtaagcaggtgaagttttagagctagaaatagcaagttaaaataaggctagtccgttatcaacttgaaaaagtggcaccgagtcggtgctttttttgaacccgggatgactgcatgtgcagctaatgc	double sgRNA for 50 kb of flagellar cluster

2sgRNAPS1	gcgcgaattcgagctcggtattgacagctagctcagtccta<br>ggtataatactagtactcacatttttcacgcatagttttagagc<br>tagaaatagcaagttaaaataaggctagtccgttatcaactt<br>gaaaaagtggcaccgagtcggtgctttttttgaacccggga<br>tgttgacagctagctcagtcctaggtataatactagtacactc<br>aagcacttcctcaggttttagagctagaaatagcaagttaaa<br>ataaggctagtccgttatcaacttgaaaaagtggcaccgag<br>tcggtgctttttttgaacccgggatgactgcatgtgcagcta<br>atgc	double sgRNA for PS1 cluster
2sgRNAPS2	gcgcgaattcgagctcggtattgacagctagctcagtccta<br>ggtataatactagtgttaatattaaaacttgcgcgttttagagc<br>tagaaatagcaagttaaaataaggctagtccgttatcaactt<br>gaaaaagtggcaccgagtcggtgctttttttgaacccggga<br>tgttgacagctagctcagtcctaggtataatactagtggggt<br>aataagcacaacatcgttttagagctagaaatagcaagttaa<br>aataaggctagtccgttatcaacttgaaaaagtggcaccga<br>gtcggtgctttttttgaacccgggatgactgcatgtgcagct<br>aatgc	double sgRNA for PS2 cluster
2sgRNAPS3	gcgcgaattcgagctcggtattgacagctagctcagtccta<br>ggtataatactagttcgatccgacaatgtattgagttttagag<br>ctagaaatagcaagttaaaataaggctagtccgttatcaact<br>tgaaaaagtggcaccgagtcggtgctttttttgaacccggg<br>atgttgacagctagctcagtcctaggtataatactagtttcac<br>gctccattcgttccagttttagagctagaaatagcaagttaaa<br>ataaggctagtccgttatcaacttgaaaaagtggcaccgag<br>tcggtgctttttttgaacccgggatgactgcatgtgcagcta<br>atgc	double sgRNA for PS3 cluster
2sgRNAPS4	gcgcgaattcgagctcggtattgacagctagctcagtccta<br>ggtataatactagtgagcagcttttaaatcggcagttttagag<br>ctagaaatagcaagttaaaataaggctagtccgttatcaact<br>tgaaaaagtggcaccgagtcggtgctttttttgaacccggg<br>atgttgacagctagctcagtcctaggtataatactagttaaac<br>attcccttccactgtgttttagagctagaaatagcaagttaaa<br>ataaggctagtccgttatcaacttgaaaaagtggcaccgag<br>tcggtgctttttttgaacccgggatgactgcatgtgcagcta<br>atgc	double sgRNA for PS4 cluster
2sgRNAFhaB	gcgcgaattcgagctcggtattgacagctagctcagtccta<br>ggtataatactagtctgcgaaaaaacaagagctcgttttaga<br>gctagaaatagcaagttaaaataaggctagtccgttatcaa<br>cttgaaaaagtggcaccgagtcggtgctttttttgaacccgg<br>gatgttgacagctagctcagtcctaggtataatactagtgcc<br>acgattacccaagagttgttttagagctagaaatagcaagtt<br>aaaataaggctagtccgttatcaacttgaaaaagtggcacc	double sgRNA for *fhaB* cluster

	gagtcggtgctttttttgaacccgggatgactgcatgtgcagctaatgc	
1sgRNAHppD	gcgcgaattcgagctcggtattgacagctagctcagtcctaggtataatactagtgaggaagaccctaagcctaagttttagagctagaaatagcaagttaaaataaggctagtccgttatcaacttgaaaaagtggcaccgagtcggtgctttttttgaacccgggatgactgcatgtgcagctaatgc	single sgRNA for *hppD*
1sgRNAHyp	gcgcgaattcgagctcggtattgacagctagctcagtcctaggtataatactagtggcgatttcttgagaagcatgttttagagctagaaatagcaagttaaaataaggctagtccgttatcaacttgaaaaagtggcaccgagtcggtgctttttttgaacccgggatgactgcatgtgcagctaatgc	single sgRNA for *hyp*
1sgRNAproFhaB	gcgcgaattcgagctcggtattgacagctagctcagtcctaggtataatactagttgctgttgttaaatgctttcgttttagagctagaaatagcaagttaaaataaggctagtccgttatcaacttgaaaaagtggcaccgagtcggtgctttttttgaacccgggatgactgcatgtgcagctaatgc	single sgRNA for promoter region of *fhaB*
20k1sgRNA	gcgcgaattcgagctcggtattgacagctagctcagtcctaggtataatactagtgccgttttaagtcagtcactgttttagagctagaaatagcaagttaaaataaggctagtccgttatcaacttgaaaaagtggcaccgagtcggtgctttttttgaacccgggatgactgcatgtgcagctaatgc	single sgRNA for 20 kb of flagellar cluster
1sgRNAO-antigen	ttgacagctagctcagtcctaggtataatactagtactagggatcaccagcgacagttttagagctagaaatagcaagttaaaataaggctagtccgttatcaacttgaaaaagtggcaccgagtcggtgctttttttgaa	single sgRNA for O-antigen cluster

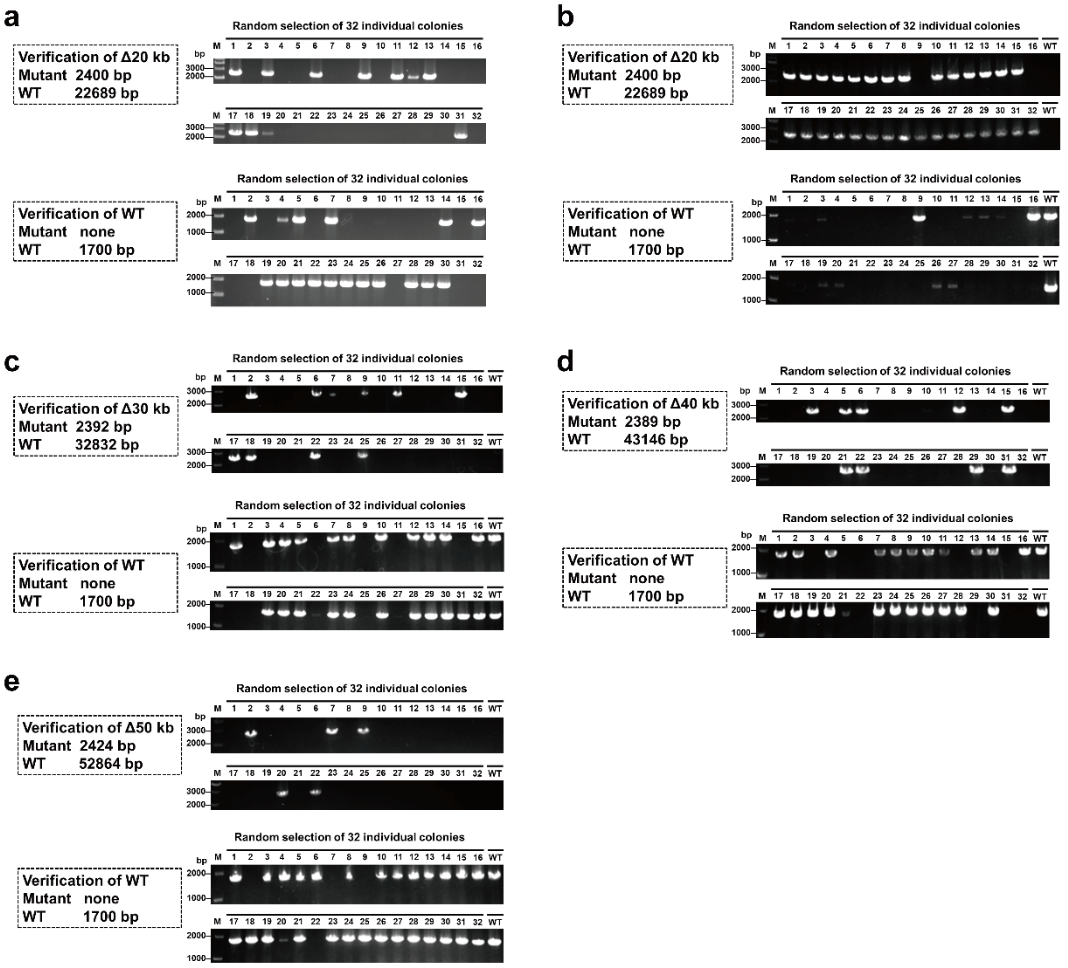

Supplementary Fig. 1. PCR verification of flagellar gene cluster deletion. Each colony was verified *via* PCR with a pair of external primers (flagella VF/flagella V20kR, flagella VF/flagella V30kR, flagella VF/flagella V40kR, flagella VF/flagella V50kR) and a pair of inner primers (flagella VF and flagella VWTR). The external primers were designed based on the upstream of upstream homologous arm and downstream of downstream homologous arm. The colonies with flagellar gene deletion (Mutant) could produce a 2.4-kb PCR product using the external primers. However, there was no PCR product for the wild-type strain using the external primers due to its theoretical size (>22 kb). The forward primer of inner primers was designed based on the upstream of the upstream homologous arm and the reverse primer on the flagellar gene cluster. The

wild-type strain yielded a 1.7-kb PCR product using the inner primers. When only the band of 2.4 kb was detected, the colony was a homozygote. When both the bands of 1.7 kb and 2.4 kb were detected, the colony was a heterozygote. **a** Deletion of 20-kb fragment with single sgRNA. **b** Deletion of 20-kb fragment with double sgRNA. **c** Deletion of 30-kb fragment with double sgRNA. **d** Deletion of 40-kb fragment with double sgRNA. **e** Deletion of 50-kb fragment with double sgRNA.

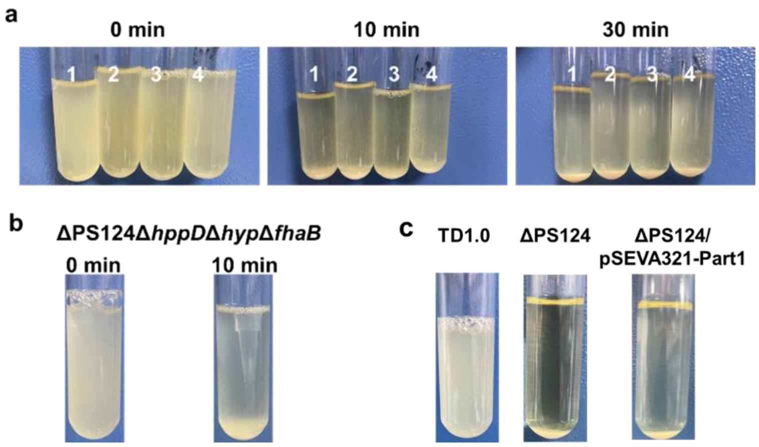

Supplementary Fig. 2. Self-flocculation of ΔPS124, ΔPS124Δ*fhaB*, ΔPS124Δ*hppD*, ΔPS124Δ*hyp*, ΔPS124Δ*hppD*Δ*hyp*Δ*fhaB*, and ΔPS124/pSEVA321-Part1. a The cultures were left standing for 0, 10 min and 30 min, respectively. 1, ΔPS124. 2, ΔPS124Δ*fhaB*. 3, ΔPS124Δ*hppD*. 4, ΔPS124Δ*hyp*. **b** The culture of ΔPS124Δ*hppD*Δ*hyp*Δ*fhaB* was left standing for 0 and 10 min, respectively. **c** Self-flocculation of TD1.0, ΔPS124 and ΔPS124/pSEVA321-Part1 after standing for 30 min.

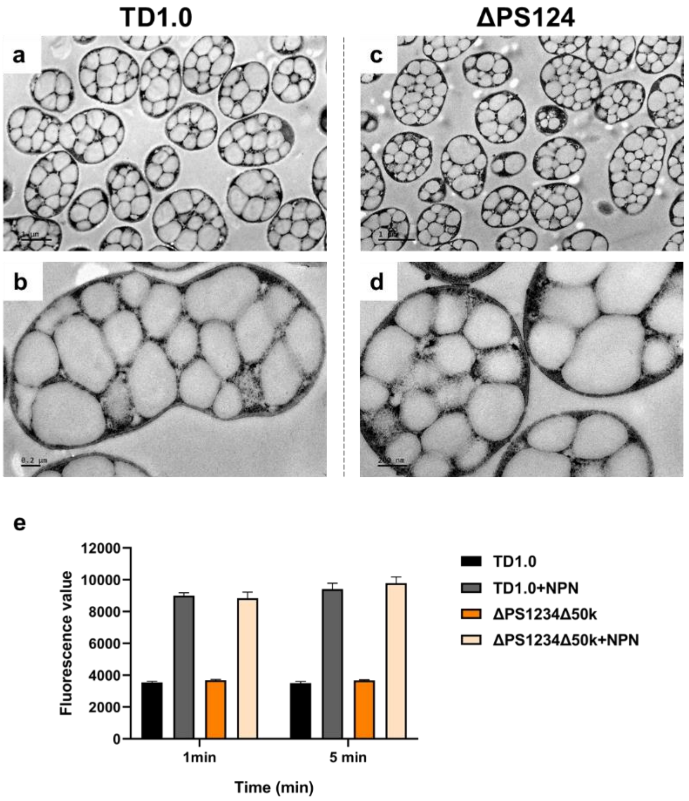

Supplementary Fig. 3. TEM images and outer membrane permeability of *H. bluephagenesis*. a-d TEM images of TD1.0 and ΔPS124. Cells were cultured in 60MMG medium for 36 h before fixation. **e** Comparison of the outer membrane permeability between TD1.0 and ΔPS1234Δ50k. Cells cultured overnight in LB60 medium were assessed using NPN method. Error bars are SD (n=3).

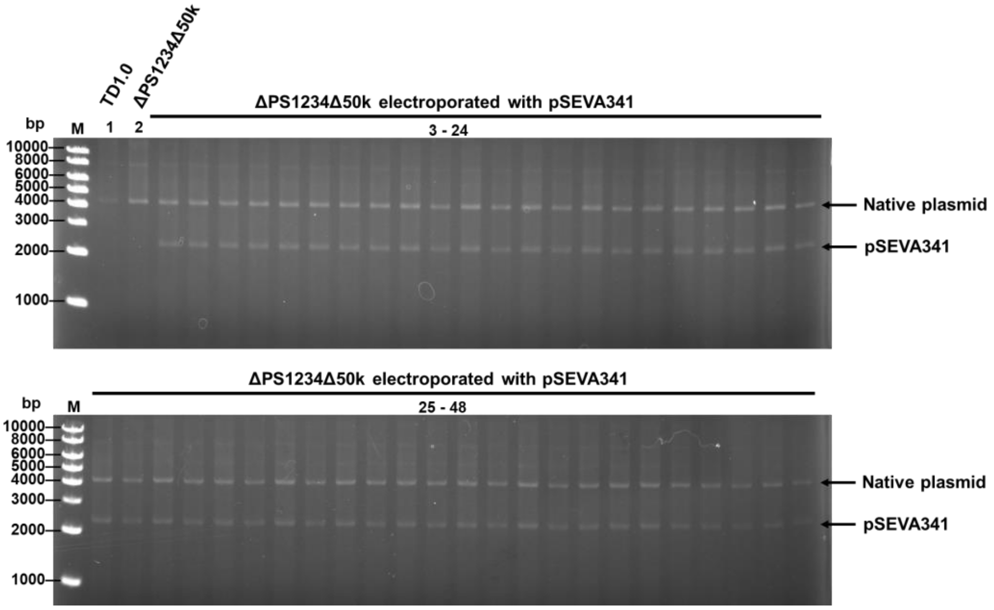

Supplementary Fig. 4. Agarose gel electrophoresis of the plasmids extracted from TD1.0 and ΔPS1234Δ50k. Lane 1， non-electrotransformed TD1.0; Lane 2, non-electrotransformed ΔPS1234Δ50k; Lane 3-48, electrotransformants of ΔPS1234Δ50k. The two black arrows indicate pSEVA341 and the native plasmid in *H. bluephagenesis*, respectively.

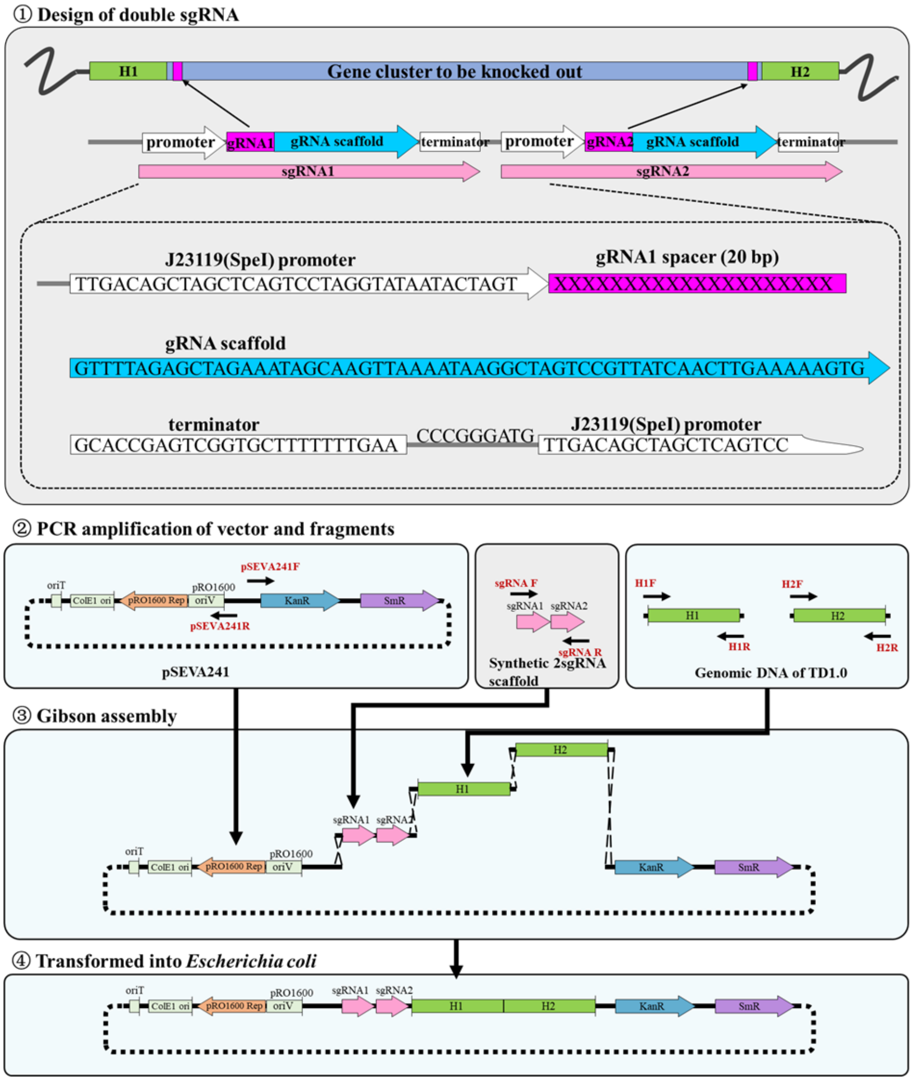

Supplementary Fig. 5. Schematic diagram for the construction of plasmids carrying double sgRNA scaffolds and homologous arms based on pSEVA241.

Supplementary References

1. Helander, I.M. & Mattila-Sandholm, T. Fluorometric assessment of gram-negative bacterial permeabilization. *J Appl Microbiol* **88**, 213-219 (2000).
2. Tian, J., Sinskey, A.J. & Stubbe, J. Kinetic studies of polyhydroxybutyrate granule formation in *Wautersia eutropha* H16 by transmission electron microscopy. *J Bacteriol* **187**, 3814-3824 (2005).
3. Simon, R. High frequency mobilization of gram-negative bacterial replicons by

the in vitro constructed Tn5-Mob transposon. *Mol Gen Genet* **196**, 413-420 (1984).

4. Zhao, H. et al. Novel T7-like expression systems used for *Halomonas*. *Metab Eng* **39**, 128-140 (2017).
5. Silva-Rocha, R. et al. The Standard European Vector Architecture (SEVA): a coherent platform for the analysis and deployment of complex prokaryotic phenotypes. *Nucleic Acids Res* **41**, D666-D675 (2013).
6. Qin, Q. et al. CRISPR/Cas9 editing genome of extremophile *Halomonas* spp. *Metab Eng* **47**, 219-229 (2018).
