# Supplementary material for: Deficiency of exopolysaccharides and O-antigen makes Halomonas bluephagenesis self-flocculating and amenable to electrotransformation
Source: Commun Biol. 2022 Jun 24;5:623. doi: 10.1038/s42003-022-03570-y (PMC9232590; doi:10.1038/s42003-022-03570-y)
Supplement: Supplementary file 3 — Description of Additional Supplementary Files [file 42003_2022_3570_MOESM3_ESM.docx]

**Description of Additional Supplementary Files**

**File name:** Supplementary Data 1
**Description:** List of flagellar genes

**File name:** Supplementary Data 2 **Description:** List of essential genes

**File name:** Supplementary Data 3 **Description:** Source data underlying the figures
